# Improvements in Outcomes and Expanding Indications for the Commando Procedure. Comment on Giambuzzi et al. Surgical Aortic Mitral Curtain Replacement: Systematic Review and Metanalysis of Early and Long-Term Results. *J. Clin. Med*. 2021, *10*, 3163

**DOI:** 10.3390/jcm11041125

**Published:** 2022-02-21

**Authors:** Lin Chen, Rashed Mahboubi, Mona Kakavand, Ozgun Erten, Eugene H. Blackstone, Douglas R. Johnston

**Affiliations:** 1 Case Western Reserve University School of Medicine, 9501 Euclid Ave., Cleveland, OH 44106, USA; 2 Department of Thoracic and Cardiovascular Surgery, Heart Vascular and Thoracic Institute, Cleveland Clinic, Cleveland, OH 44195, USA; mahbour@ccf.org (R.M.); kakavam@ccf.org (M.K.); erteno@ccf.org (O.E.); blackse@ccf.org (E.H.B.); johnstd3@ccf.org (D.R.J.)

We read with interest the authors’ review and metanalysis of the Commando procedure in “Surgical Aortic Mitral Curtain Replacement: Systematic Review and Metanalysis of Early and Long-Term Results” [1]. Aortic and mitral valve replacement with reconstruction of the intervalvular fibrous body (subaortic curtain), pioneered by Bruce Lytle and David Tirone, was named “Commando” due to the technically challenging nature of this procedure [2,3]. There have only been a handful of contemporary publications reporting the outcomes of the procedure, limited to high-volume tertiary cardiac surgery centers. Given the limited data, we commend the authors for their review and analysis of the literature.

We would like to draw attention to the authors’ methodology. We caution the readers that the authors included four studies with overlapping patient populations in their analysis, despite removing “updated series” in their Methods section [3,4,5,6]. Although unconventional, this presents an opportunity to discuss the improvements in outcomes of the procedure over the past several decades, as demonstrated by the two studies from Toronto General Hospital. David and colleagues presented their initial cohort of patients from 1985 to 1996 and the updated series from De Oliveira and colleagues included patients from 1985 to 2002 [3,4]. Between these two studies, operative mortality decreased from 16% to 10% and 5-year survival increased from 56% to 71%.

In the two publications from our institution, Elgharably reported outcomes of the Hemi-Commando procedure from 2010 to 2017, and Navia reported the outcomes of the Commando and Hemi-Commando procedures from 1988 to 2017 [5,6]. We would like to draw attention to the differences between these two procedures. Despite the similarity in verbiage, the Hemi-Commando procedure, developed by Jose Navia, involves the implant of an aortic valve alloagraft with its attached anterior mitral valve cusp and mitral valve repair, with preservation of its posterior cusp. The Commando procedure instead reconstructs the intervalvular fibrous body with a pericardial patch and replaces the aortic and mitral valve. When evaluating the outcomes of the procedures, the differences in disease progression should be taken into account. The Hemi-Commando is only indicated for patients with invasive infectious endocarditis limited to the base (atrial zone) of the anterior mitral cusp, without involvement of the free edge (coaptation zone) receiving the primary and secondary chordae, but the Commando procedure is performed for patients with more extensive disease involving the entire intervalvular fibrous body. Currently, the case series of seven patients from Navia and colleagues is the largest series of Hemi-Commando procedures, but lacks the power to evaluate differences in outcomes.

With these procedures performed more frequently at high-volume centers, improving outcomes over time have encouraged the dissemination of the Commando procedure to noninfectious indications, namely radiation-associated cardiac disease and severe mitral annular calcification in the absence of radiation. We eagerly anticipate more data on the outcomes of Commando and Hemi-Commando procedures from other centers.
